# Laparoscopic Infrahepatic Inferior Vena Cava Clamping During Liver Resection — a Feasibility Study

**DOI:** 10.1007/s11605-023-05593-0

**Published:** 2023-01-19

**Authors:** Patrick Téoule, Niccolo Schmidt, Erik Rasbach, Emrullah Birgin, Christoph Reissfelder, Nuh N. Rahbari

**Affiliations:** 1 Department of Surgery, Medical Faculty Mannheim, Universitätsmedizin Mannheim, Heidelberg University, Theodor-Kutzer-Ufer 1-3, 68167 Mannheim, Germany; 2 DKFZ-Hector Cancer Institute at University Medical Center Mannheim, Mannheim, Germany

**Keywords:** Hepatectomy, Central venous pressure, Minimal invasive, Blood loss, Outcome

## Introduction

Intraoperative hemorrhage is a major predictor of perioperative outcomes after hepatectomy. Infrahepatic inferior vena cava (IVC) clamping has already been proven as a safe and effective technique to reduce intraoperative blood loss (BL) in open liver resection (OLR).^1^ However, its effectiveness and safety in laparoscopic liver resection (LLR) remains unclear. Therefore, it was the objective of this study to evaluate the perioperative outcomes and feasibility of IVC-clamping in LLR at a European tertiary-center.

## Methods

Patients undergoing LLR with intraoperative IVC-clamping at our department were identified from a prospective database (August 2020–August 2021).

**Fig. 1:**
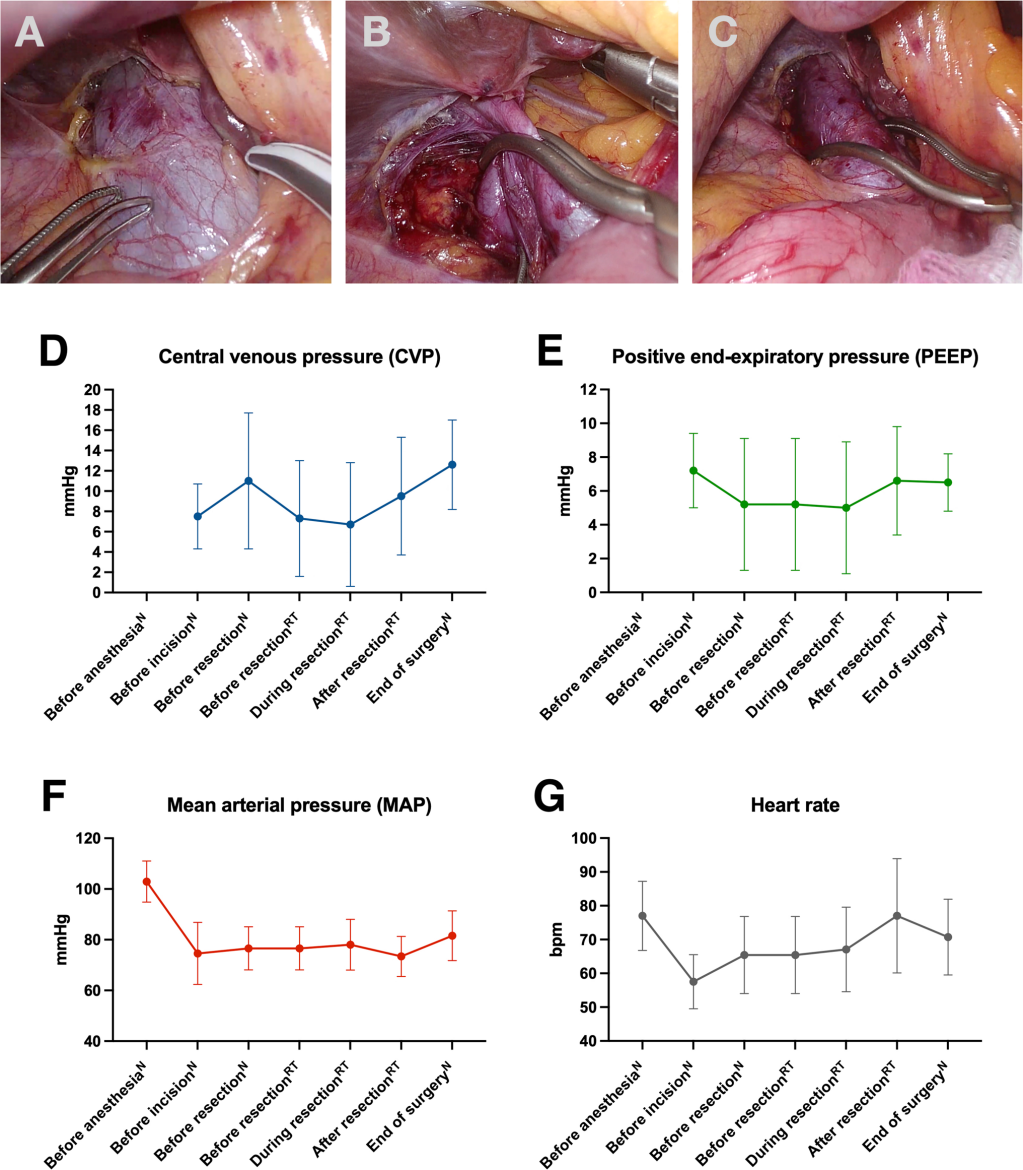
**a** Infrahepatic IVC-clamping during LLR. (**A**) Laparoscopic sight of the IVC and preparation below the hepatoduodenal ligament; (**B**) Positioning of the Bulldog clamp before resection; (**C**) Release of the clamp after resection. **b** Intraoperative hemodynamic parameters and positive end-expiratory pressure. N, CVP measured in neutral position; RT, CVP measured in reverse Trendelenburg position; values are mean (s.d.)

To gain access to the IVC, the liver was lifted up, and the IVC was dissected right below the hepatoduodenal ligament above the level of the left renal vein. Once dissected, the IVC was clamped (Fig. 1a). Initially, the IVC was clamped partially to confirm the patients’ hemodynamic stability in consultation with the anesthesiologist. If tolerated, the clamp was pushed forward and closed consecutively. Parenchymal transection was carried out under low central venous pressure (CVP) and (i) CO_2_ pneumoperitoneum of at least 15 up to 18 mmHg, (ii) reversed Trendelenburg position, and (iii) intermittent Pringle maneuvers (max. 15 min of ischemia followed by 5 min of reperfusion).

Demographics and outcomes were analyzed using descriptive statistics.

## Results

Ten patients underwent LLR with IVC-clamping (Table 1). In all patients, IVC-clamping was established without any complication. Intraoperative hemodynamic parameters are summarized in Fig. 1b. Mean CVP during resection was 6.7 mmHg (s.d.: ± 6.1 mmHg). Temporary hemodynamic instability (positive shock-index) occurred in five patients and ceased spontaneously. MAP trajectory in Fig. 1b (F) indicates that MAP was never below 60 mmHg. Median operating time was 258 min (interquartile range (*IQR*), 212–302 min) and median BL during transection was 320 mL (*IQR*, 170–740 mL). In three patients, minor air embolisms were detected, but without any clinical consequences and were treated by intermittent hyperventilation. Functional recovery was achieved after a median of 4 days (*IQR*, 2–4 days). Median length of stay was 6 days (*IQR*, 5–7 days). Two patients developed clinically relevant complications (Clavien-Dindo ≥ III), both presented with post-hepatectomy bile leakage and needed to be readmitted. No mortality occurred within 90 days after surgery.

**Table 1:** Patient characteristics and outcomes

	*N* (%) or median (*IQR*)
Age (years)	68 (61–76)
Sex ratio, male:female	7:3
BMI (kg/m^2^)	28 (26–29)
ASA	
I	1 (10)
II	4 (40)
III	5 (50)
Diabetes	4 (40)
Diagnosis	
Primary liver malignancy	7 (70)
Hepatocellular carcinoma	5 (50)
Intrahepatic cholangiocarcinoma	2 (20)
Secondary liver malignancy	3 (30)
Colorectal liver metastasis	1 (10)
Other	2 (20)
Cirrhosis	4 (40)
IWATE Criteria	8 (6 – 9)
Previous abdominal surgery	6 (60)
Extent of resection	
Major hepatectomy^§^	1 (10)
Minor hepatectomy	9 (90)
Surgical procedure	
(Extended) right hemihepatectomy	1 (10)
Right posterior sectionectomy	2 (20)
Other anatomic resection $\le$ 2 segments	5 (50)
Non-anatomical/ atypical resection	2 (20)
Additional procedures	
Cholecystectomy	8 (80)
Hiliar lymphadenectomy	2 (20)
Operating time (min)	258 (212–302)
Transection time (min)	114 (84–186)
Duration of portal triad clamping (min)	67 (51–79)
Duration of IVC clamping (min)	63 (20–78)
Blood loss (total) (mL)	620 (360–1000)
Blood loss (during transection) (mL)	320 (170–740)
Blood loss per transection area (mL/cm^2^)	4.4 (1.9–9.0)
Transection surface area (cm^2^)	80 (67–106)
Infused crystalloid fluids (mL)	2000 (1350–2680)
Norepinephrine dose (µg/min total operating time)	6.6 (5.4–8.1)
Norepinephrine dose (µg/min transection time)	6.9 (5.8–9.3)
Post-operative complications	
< Grade III	3
≥ Grade III	2
Comprehensive Complication Index^†^	4.4 (0.0–8.7)
Post-hepatectomy complications^a^	
Post-hepatectomy hemorrhage	0
Posthepatectomy liver failure^b^	2 (20)
Posthepatectomy bile leackage^c^	2 (20)
Specific complications^a^	
Pneumonia	1 (10)
Wound infection	1 (10)
Renal failure	1 (10)
Invasive interventions^a^	
Endoscopic intervention	2
Radiologic drainage (CT or ultrasound-guided)	3
Readmission	2 (20)
Time to functional recovery^d^ (d)	4 (2–4)
Length of IMC stay (d)	1 (0–1)
Postoperative length of stay (d)	6 (5–7)

Other = one anal and one laryngeal carcinoma

^§^Resection of more than two anatomical segments;

^a^Multiple answers are possible;

^b^One with Grade A and one with Grade B defined by International Study Group of Liver Surgery(ISGLS);

^c^Grade B defined by ISGLS;

^d^Functional recovery according to van Dam et al.^2^

## Discussion

The second international consensus conference for LLR held in Morioka (2014) recommended a low CVP during LLR to achieve a reduction of BL. A possible reduction of BL with low CVP by restrictive fluid administration comes at the cost of a higher risk of hemodynamic instability in case of major BL.^1^ A study on OLR revealed that IVC-clamping could be used as a potential alternative to conservative CVP-lowering to reduce BL.^1^ Despite this proven effectiveness, IVC-clamping in LLR has been poorly explored and not been performed routinely. Recently, the evidence of LLR with IVC-clamping has only been reported in one retrospective study.^3^ However, within this study, authors neither defined the time period of reported morbidity nor provided data on CCI or functional recovery. The overall BL in our study is higher than expected, compared to other studies. This could be biased by our small study group or could be explained by the complexity of the performed operations (median IWATE score of eight). Furthermore, the presence of cirrhosis in 40% of patients might have contributed to the slightly higher BL. Within a RCT on the impact of IVC-clamping on BL during OLR, a significantly higher rate of post-operative pulmonary embolism (PPE) was observed.^1^ Therefore, uncertainty about the impact of IVC-clamping during LLR exists. However, a recent meta-analysis confirmed that IVC-clamping is not associated with increased incidence of PPE in OLR.^4^ In this study, we did not observe any case of PPE. Compared to other studies performing LLR without IVC-clamping, our results showed no potential risk or increased morbidity and mortality.^5,6^

The present study provides initial data on the feasibility and safety of IVC-clamping during LLR and justify future trials to evaluate the effectiveness of this technique to reduce CVP and BL during LLR.
